# Thermal and Thermoelectric Transport in Highly Resistive Single Sb_2_Se_3_ Nanowires and Nanowire Bundles

**DOI:** 10.1038/srep35086

**Published:** 2016-10-07

**Authors:** Ting-Yu Ko, Muthaiah Shellaiah, Kien Wen Sun

**Affiliations:** 1 Department of Applied Chemistry, National Chiao Tung University, 1001 University Road, Hsinchu, 30010, Taiwan; 2 Department of Electronics Engineering, National Chiao Tung University, 1001 University Road, Hsinchu, 30010, Taiwan

## Abstract

In this study, we measured the thermal conductivity and Seebeck coefficient of single Sb_2_Se_3_ nanowires and nanowire bundles with a high resistivity (σ ~ 4.37 × 10^−4^ S/m). Microdevices consisting of two adjacent suspended silicon nitride membranes were fabricated to measure the thermal transport properties of the nanowires in vacuum. Single Sb_2_Se_3_ nanowires with different diameters and nanowire bundles were carefully placed on the device to bridge the two membranes. The relationship of temperature difference on each heating/sensing suspension membranes with joule heating was accurately determined. A single Sb_2_Se_3_ nanowire with a diameter of ~ 680 nm was found to have a thermal conductivity (k_NW_) of 0.037 ± 0.002 W/m·K. The thermal conductivity of the nanowires is more than an order of magnitude lower than that of bulk materials (k ~ 0.36–1.9 W/m·K) and highly conductive (σ ~ 3 × 10^4^ S/m) Sb_2_Se_3_ single nanowires (k ~ 1 W/m·K). The measured Seebeck coefficient with a positive value of ~ 661 μV/K is comparable to that of highly conductive Sb_2_Se_3_ single nanowires (~ 750 μV/K). The thermal transport between wires with different diameters and nanowire bundles was compared and discussed.

Recovering waste heat and converting it into electrical energy with thermoelectric generators is an effective measure against global warming and environmental impact of climate change. Thermoelectric devices that are noiseless (no moving parts), inherently reliable without any maintenance, and environmentally friendly have attracted increasing attention^1^. Research on thermoelectric materials has strongly increased over the recent years because of their high application potential. A previous study reported that fuel efficiency can be improved by replacing the alternator in cars with a thermoelectric generator mounted on the exhaust stream^2^.

Thermoelectric power generation is based on the Seebeck effect, in which a temperature gradient is converted into an electric current. The applications of bulk thermoelectrics in cost-effective devices are limited by their low efficiency. Thus, new materials with improved thermoelectric power efficiency need to be developed. Dresselhaus *et al*.^3^,^4^,^5^,^6^ predicted that thermoelectric efficiency can be significantly enhanced through nanostructural engineering. This proposal led to experimental efforts to demonstrate nanostructured systems with improved efficiency^7^,^8^,^9^,^10^,^11^,^12^,^13^. Recently, nanowires (NWs) have garnered increasing attention in various applications because of their distinct properties, such as high surface area and quantum confinement. One-dimensional nanostructures are specifically attractive for energy conversion applications where photons, phonons, and electrons are important. The Bohr exciton radius, and phonon and electron mean free paths are on the same length scales as NW size adjustment. In thermoelectric power generation, the NW diameter can effectively scatter phonons, thereby reducing thermal conductivity and enhancing the thermoelectric figure of merit (ZT)^14^,^15^,^16^,^17^,^18^,^19^,^20^,^21^.

V_2_VI_3_-type semiconductors possess band gap energies ranging from 2.2 eV [e.g., Sb_2_S_3_] to 0.21 eV [e.g., Bi_2_Te_3_]. Sb_2_Se_3_ is a layered semiconductor with an orthorhombic crystal structure; this material has been studied because of its switching effects^22^, as well as favorable photovoltaic and thermoelectric properties^23^,^24^. Thin polycrystalline films of Sb_2_Se_3_ semiconductors have been commonly used as absorbers to fabricate cost-effective solar cells and Hall effect devices^25^. Sb_2_Se_3_ films are widely applied in optical materials, thermoelectric cooling, and power conversion devices because of their high thermoelectric power^26^. Over the past two decades, many methods have been developed to prepare Sb_2_Se_3_ nanostructures (nanotubes, NWs, and nanobelts). Several studies have analyzed the electrical properties and photoresponse of Sb_2_Se_3_ nanomaterials^27^,^28^,^29^,^30^,^31^,^32^,^33^, but, detailed studies on the thermoelectric properties and thermal conductivity of Sb_2_Se_3_ nanostructures remain lacking.

In the present study, microfabricated devices hybridized with individual Sb_2_Se_3_ NWs and NW bundles were used to measure the thermal transport properties of NWs. The thermal conductivity of the NWs was more than an order of magnitude lower than that of bulk materials in previous measurements. The measured Seebeck coefficient is about two-fold lower than that of the bulk. We also investigated the effects of NW diameters and van der Waals interface between NWs on thermal conductivity.

## Nanowire synthesis

**Figure 1:**
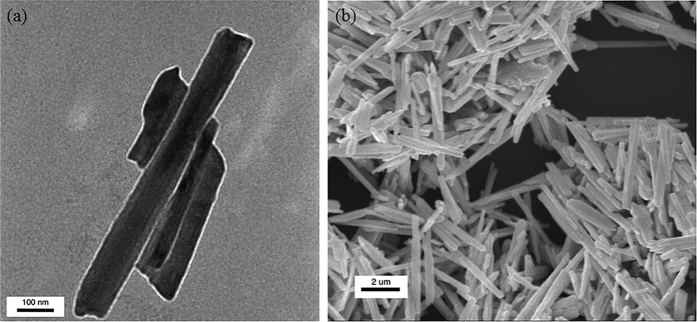
TEM and SEM images of the fabricated Sb_2_Se_3_ NWs at 100 °C and 200 °C, respectively.

**Figure 2:**
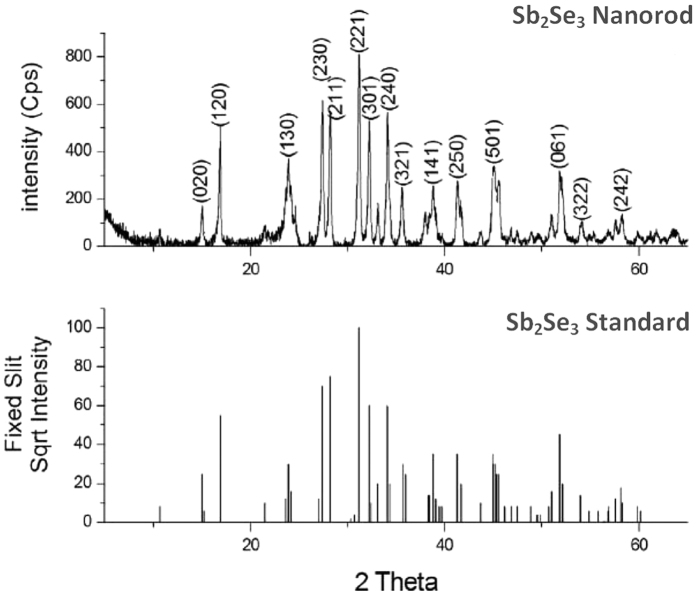
XRD patterns of the Sb_2_Se_3_ NWs and Sb_2_Se_3_ standard (Joint Committee on Powder Diffraction Standards (JCPDS) 15-0861).

**Figure 3:**
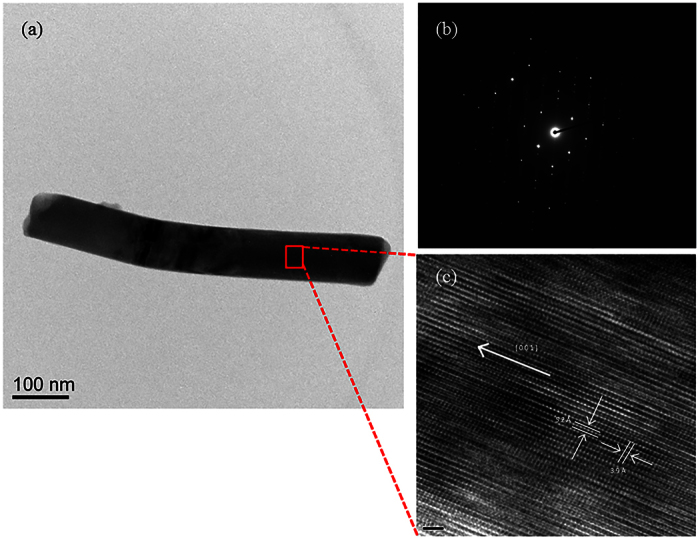
(**a**) TEM image (**b**) SAED pattern and (**c**) HRTEM images of an individual nanowire and the corresponding crystal planes.

Sb_2_Se_3_ NW samples with a high-quality single-crystalline property were produced from a single source precursor, namely, Sb[Se_2_P(O^i^Pr)_2_]_3_. These materials have already been studied and were demonstrated elsewhere^34^,^35^,^36^ by carefully adjusting the experimental parameters. Transmission and Scanning electron microscope images (Fig. 1) of the fabricated NW clusters annealed at different temperatures indicate that the NWs exhibit an average diameter from 100 nm to 300 nm and a length of 3–10 μm. NWs with wide distributions in diameters can be fabricated by controlling the annealing temperature from 100 °C to 200 °C. Details on the preparation of the precursor, synthesis of Sb_2_Se_3_ NWs, and morphology characterization can be found in the Supplementary Information. The composition of the NWs was determined to be Sb_2_Se_3_ with a ratio of 1:1.5 as determined by powder X-ray diffractometry (Fig. 2) and energy-dispersive spectroscopy (EDS). HRTEM images (Figs 3, S1 and S2) of individual NWs reveal that these materials comprise single crystals and without dislocations.

### Microdevice fabrication

**Figure 4:**
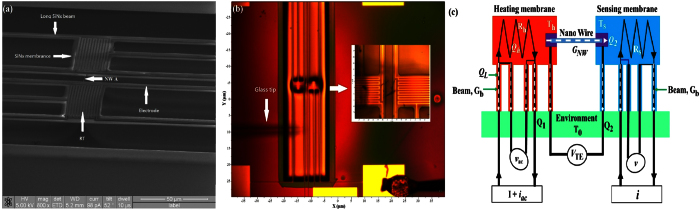
(**a**) SEM micrograph of a microdevice for thermal property measurements of a single NW with a diameter of 227 nm (NW A). Two Cr/Au coils serve as resistance thermometers (RT) on each membrane. (**b**) Optical image showing a single NW was placed across the membranes using a nanomanipulator. (**c**) Schematic diagram and thermal resistance equivalent circuit of the measurement.

Suspended microdevices fabricated on SiN_x_/SiO_2_/Si through electron beam lithography, photolithography, metallizations, and etching were employed to measure thermal conductivity. Details on devices fabrication can be found in refs 10,14,15,37 and 38. Figure 4(a) shows that the device consists of two adjacent 24 μm × 40 μm low-stress SiN_x_ membranes suspended with 300 μm long SiN_x_ beams. A 50 nm-thick Cr/Au resistive thermometer coil was designed on each membrane, which acts as both a heater to heat up the heating membrane and a thermometer to measure the temperature of the sensing membrane. As shown in Fig. 4(a,b), an individual Sb_2_Se_3_ NW with a diameter of 227 nm (NW A) was positioned between two membranes using of a nanomanipulator. The device was placed in a vacuum chamber (10^−7^ Torr) at room temperature to avoid thermal radiation loss, making Sb_2_Se_3_ NW the only pathway to transport heat between the heating and sensing membranes. A detailed description of the measurement techniques and uncertainty analysis can be found in refs 14,15,17,39 and 40.

### Heat transfer model of microdevice

The application of a direct current voltage to the Cr/Au coil on the heating membrane (with a resistance R_h_) caused joule heat generation and a consequent temperature rise ΔT_h_ (ΔT_h_ = T_h_ − T_0_) from the thermal bath temperature T_0_. A certain amount of heat generated by the Cr/Au coil on the heating membrane was transported to the sensing membrane (with a resistance R_s_), either through an individual Sb_2_Se_3_ NW or NW bundle with negligible heat loss. Heat conduction via the Sb_2_Se_3_ NWs or NW bundles increases the temperature of the sensing membrane (ΔT_s_ = T_s_ − T_0_). The temperature difference between the heating and sensing membranes was controlled within 12 degree to prevent heat radiation. Using a simple heat transfer model^37^ (Fig. 4(c)), we can estimate the thermal conductance of the NWs or NW bundles, G_NW_, and the suspending beams, G_b_, by using the following equations: 
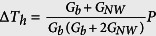
, 
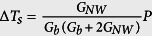
, 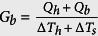, and 
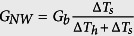
, where P is the heat power applied to the heating membrane R_h_, and Q_h_ and Q_b_ are the joule heat generated in the heating membrane and metallic wire of the suspending beams, respectively. Under thermal steady state condition, the thermal conductivities through a single Sb_2_Se_3_ NW or a NW bundle can be retrieved using the above equations by measuring the temperature coefficients of resistance from both membranes, ΔT_h_, ΔT_s_, and joule heat power P.

## Results and Discussions

**Figure 5:**
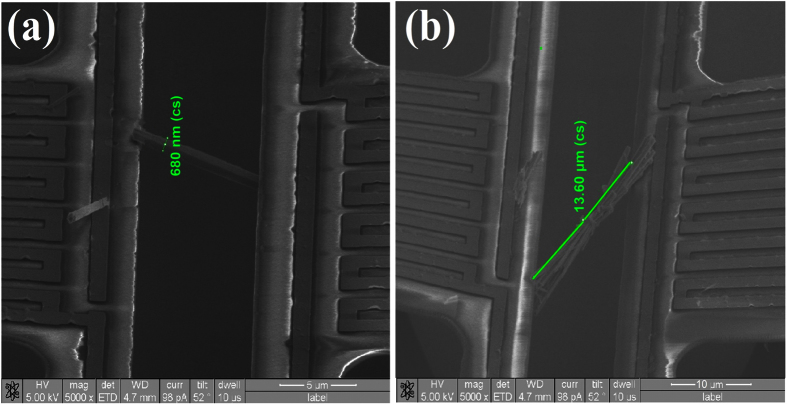
SEM images of single NWs across the membranes with diameters of (**a**) 680 nm (NW B) and (**b**) NW bundle (NW C).

Figures 4(a) and 5 shows the SEM images of three NW samples, namely, NW A, NW B, and NW C, which bridge the heating and sensing membranes. NWs A (Fig. 4(a)) and B (Fig. 5(a)) are both single NWs; NW A has a length of 11 μm and a diameter of 227 nm, respectively, whereas NW B has a length of 8.9 μm and a diameter of 680 nm. NW C (Fig. 5(b)) is composed of six to seven closely packed NWs. Each NW in the bundle has a diameter of approximately 100 nm and an average length of 13.6 μm, which makes the total diameter of the NW bundle approximately 750 nm. In the following, we use the thermal conductance measurements of sample NW B as an example. The determination of thermal conductance for the remaining NWs basically follows the same procedure as that for NW B.

**Figure 6:**
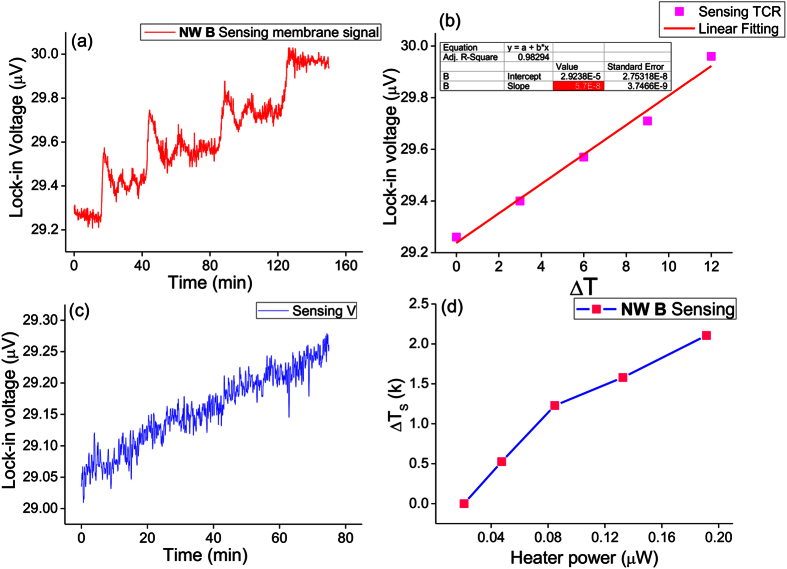
Voltage signal on the sensing membrane as a function of (**a**) time and (**b**) temperature difference. In (**a**), temperature of the sensing membrane was varied every 20 mins with an increment step of 3 °C. Temperature coefficient of resistance (TCR) was determined by measuring the slope of the cure in (**b**). (**c,d**) show the voltage signal and temperature change on the sensing membrane as a function of time and heater power, respectively, when the NW B is placed across the membranes and the heating membrane is joule-heated by applying an electrical current from 4 μA to 12 μA.

**Figure 7:**
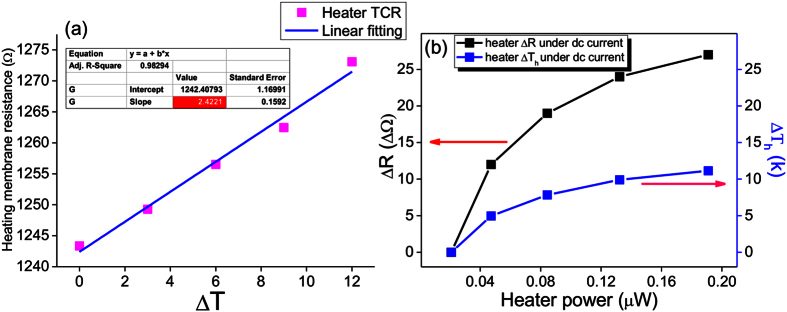
(**a**) Heating membrane resistance as a function of temperature changes and (**b**) changes in heating membrane resistance (ΔR) and temperature (ΔT_h_) as a function of joule heating power by applying an electrical current from 4 μA to 12 μA. In (**a**), temperature of the heating membrane was varied every 20 mins with an increment step of 3 °C. TCR was determined by measuring the slope of the cure in (**a**).

Figure 6(a) shows the voltage change on the sensing membrane when the membrane temperature is slowly increased by joule heating, with an increasing step of 3 K for every 20 min. The temperature coefficient of resistance (TCR) β (= 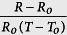, R_o_: resistance at temperature T_o_) with a value of 5.7 × 10^−8^ K^−1^ for the sensing membrane was determined by fitting the slope of the curve (Fig. 6(b)). With the NW B placed across the membranes, the voltage signal and temperature change (ΔT_s_) on the sensing membrane were recorded when the heating membrane was joule-heated by applying an electrical current within the range of 4–12 μA (Fig. 6(c,d)). We measured the resistance and change in resistance (ΔR) of the heating membrane as functions of temperature change and input joule heat (Fig. 7(a,b)), respectively. On the basis of the combined results from Fig. 7(a,b), the changes in heating membrane temperature (ΔT_h_) as a function of input joule heat was determined (Fig. 7(b)). By reading the data from Figs 6(d) and 7(b), at a joule heat power of 4.67 × 10^−8^ W, the temperature changes on the heating and sensing membranes are ΔT_h_ = 4.95 K and ΔT_s_ = 0.52 K, respectively. Inputting these values in the above equations and assuming that thermal contact resistance was negligible^14^, we find that the thermal conductance and thermal conductivity of NW B are G_NW_ = 1.522 × 10^−9^ W/K and k_NW_ (G_NW_ × L/A) = 0.037 W/m·K, respectively. The measured thermal conductivity, k_NW_, is composed of contributions from both the electronic part (k_e_) and the phonon part (k_ph_). It is written as: k_NW_ = k_e_ + k_ph_. The electronic contribution to the thermal conductivity of NWs can be calculated using the Wiedemann-Franz law: k_e_ = σL_w_T, where L_w_ is the Lorenz number. We estimated the k_e_ ~ 1.9 × 10^−9^ W/m·K for σ = 4.37 × 10^−4^ S/m using a Lorenz number L_w_ = 1.45 × 10−8 V^2^/K^2^ 41 at T = 300 K, which is significantly lower than the phonon thermal conductivity k_ph_. Therefore, the measured NW thermal conductivity is governed by the lattice thermal conductivity.

Since the electrical resistance of the heating and sensing membranes was determined by a four-point probe technique, the maximum uncertainty of the electrical resistance values is less than 0.225%. The uncertainty in the thermal conductivity arises from measurement errors in voltage, current, temperature, and dimensions of the membranes. The width and length of the membranes are measured with a scanning electron microscope. The metal film thickness of membranes is measured with a Dektak surface profiler (with a resolution of 0.1 nm). The errors produced by the dimension measurements are estimated to be less than 3%. The total error in the thermal conductivity value is estimated to be within 5.5%. Therefore, the thermal conductivity of the Sb_2_Se_3_ NWs that we measured is 0.037 ± 0.002 W/m·K. The measured thermal conductivity is more than an order of magnitude lower than those of bulk materials in previous measurements (~0.36–1.9 W/m·K)^39^. Mehta *et al*.^40^ reported a thermal conductivity of less than 1 W/m·K on individual highly conductive Sb_2_Se_3_ nanocrystals (with an electrical conductivity of ~ 3 × 10^4^ S/m), which was about 2.5-fold lower than that of bulk. Our results suggested that the thermal conductivity of the NWs was only raised by nearly two orders of magnitude even when the electrical conductivity of the NWs was increased by 8 orders of magnitude.

**Figure 8:**
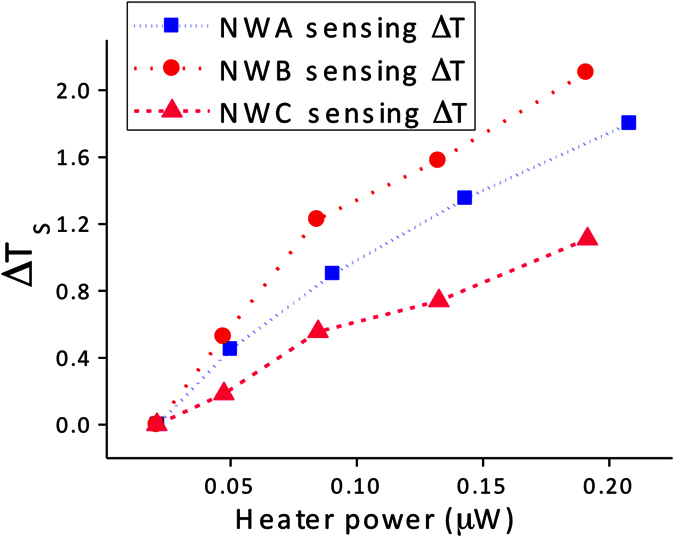
Changes in the sensing membrane temperature (ΔT_s_) of NW A, NW B, and NW C as a function of joule heating power when an electrical current from 4 μA to 12 μA was applied to the heating membrane.

Thermal conductance and thermal conductivity of NWs A and C were also determined following the same experimental procedure. The temperature changes on the sensing membranes of NWs A and C as a function of joule heat power are displayed in parallel with NW B (Fig. 8). The temperature increase on the sensing membrane of NW A, which has a diameter three times narrower than NW B, is lower than that of sample B. This observation indicates that less heat is transferred when a single NW with a smaller diameter is placed across the membranes. Therefore, the thermal conductivity of a NW decreases as its diameter is reduced. Although it has been suggested that surface roughness interacts with a broadband spectrum of phonons in NWs, resulting in decreased thermal conductivity due to frequency-dependent scattering. For example, rough Si NWs prepared by the aqueous electroless etching method exhibited five- to eight-fold lower in thermal conductivity in compared to the smooth surface Si NWs with similar diameters^42^,^43^. More recently, Liu and co-workers^44^ performed non-equilibrium molecular dynamics calculations to investigate thermal transport in crystalline-core amorphous-shell Si NWs. They found that higher rates of diffusion reflection or backscattering of phonons in the amorphous region could possibly lead to reduction in thermal conductivity. However, our Sb_2_Se_3_ NWs prepared at different temperatures are similar in surface roughness and crystallinity as measured from HRTEM images (Figures S1 and S2 in the Supplementary Information). Therefore, the possibility of lower thermal conductivity due to surface roughness effects can be ruled out. Considering that phonon boundary scattering considerably reduces the thermal conductivity of a NW, we deduce that the enhanced boundary scattering caused by the size effect suppresses phonon transport through the Sb_2_Se_3_ NWs^15^,^45^.

Interestingly, the NW bundle (NW C) gives the lowest temperature rise on the sensing membrane among the three samples, even though it has the largest total cross section. Although thermal conductivity measurements made on an individual 120 nm-diameter NW (Figures S3 and S4 in the Supplementary Information) showed smaller temperature rises on the sensing membrane (ΔT_s_) and less heat transferred compared to NW A and NW B. However, amount of heat transferred to the sensing membrane through the bundle (NW C) was still lower than that of a single 120 nm-diameter NW. In the NW bundle, the weak van der Waals adhesion energy between NWs results in a low possibility for phonon transmission through the van der Waal interfaces^46^,^47^. Therefore, phonons in a NW bundle are scattered at the interface, leading to a phonon mean free path that is the same or less than that of a single free-standing NW. In general, nanostructure ensembles have lower thermal conductivity than a single nanostructure because of the presence of van der Waals interactions^48^,^49^,^50^,^51^. Hone *et al*.^48^ found that the thermal conductivity of carbon nanotubes bundle is significantly lower than that of a single free-standing carbon nanotube. Therefore, the decreased thermal transport of our NW bundle is attributed to the phonon scattering at the van der Waals interfaces.

**Figure 9:**
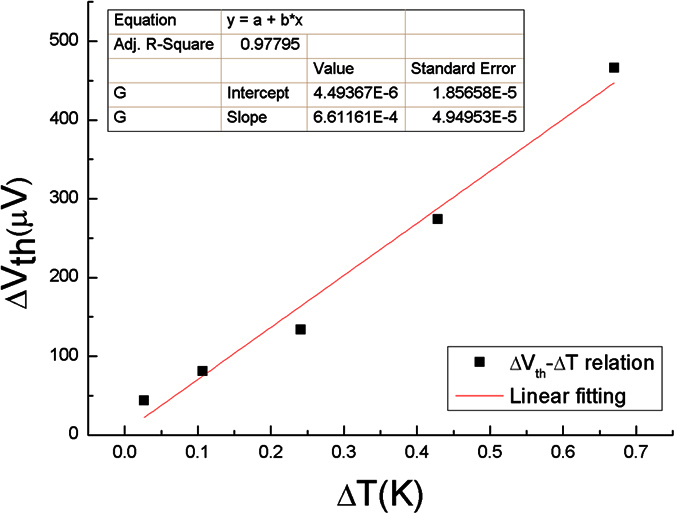
Thermal voltage changes are plotted as a function of temperature difference across the NW B. The slope of the cure determines the Seebeck coefficient.

The Seebeck coefficient S of the NW B was obtained using the 2ω technique with above microdevices. For this method, an AC current at frequency ω was applied, which produces joule heating in the membrane with a frequency of 2ω. The heat generated causes a temperature oscillation that passes through to the NW. Using the four-probe technique, the temperature difference ΔT(2ω) across the NW can be measured, along with the voltage drop ΔV(2ω). Thus, a positive Seebeck coefficient of the NW B was obtained from S = ΔV(2ω)/ΔT(2ω) ≈ 661 μV/K (Fig. 9). This value is comparable to that of highly conductive NWs (≈ −750 μV/K)^41^, but is about a factor of two lower than their bulk counterparts^52^. Although the Seebeck coefficient is predicated to increase with reduced dimensions of the material due to a higher density of states near the Fermi level^3^,^8^,^53^. However, the higher surface defects and trap charge states in NWs may lead to lower Seebeck coefficient because of the reduced electronic mean free path l_o_ by the increased scattering^54^. Further analysis of the dominant cause of the Seebeck coefficient reduction will be quite useful. The figure of merit ZT of the NWs, which is written as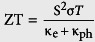, can be determined by knowing the electric conductance (σ), Seebeck coeffieient (S), and thermal conductivity (k_e_, k_ph_). When values of the measured σ (4.37 × 10^−4^ S/m) and S (661 μV/K) of the NWs are incorporated into above expression, we get a ZT value of approximately 1.55 × 10^−6^ at 300 K. Since k_e_ is rather small in compared to k_ph_, therefore, the electrical conductivity can be increased without significantly affecting the total thermal conductivity (k_e_ + k_ph_). Also the reduced dimensionality did not significantly affect the Seebeck coefficient.

## Conclusions

In Summary, thermoelectric properties of individual solvothermally prepared single-crystalline Sb_2_Se_3_ NWs and NW bundles were investigated using a microfabricatde suspended device at room temperature. We demonstrated microdevices and techniques that can measure the thermal properties of highly resistive single Sb_2_Se_3_ NWs and NW bundles. The relationship of temperature difference on each heating/sensing suspension membranes with joule heating was accurately determined. A single Sb_2_Se_3_ NW with a diameter of 680 nm was found to have a thermal conductivity of 0.037 ± 0.002 W/m·K. The thermal conductivity of the NWs is more than an order of magnitude lower than that of bulk materials. The thermal conductivity of a NW decreases as its diameter is reduced. The NWs in the NW bundles interact through van der Waals interactions, which reduce thermal transport. A positive Seebeck coefficient of ≈ 661 μV/K was obtained, which is comparable to that of highly conductive NWs, but is about a factor of two lower than bulk due to the increased scattering of higher surface defects and charge trap states in NWs. These excellent thermal properties indicate that Sb_2_Se_3_ NWs are promising candidates for thermoelectrical energy conversion nanomaterials.

## Additional Information

**How to cite this article**: Ko, T.-Y. *et al*. Thermal and Thermoelectric Transport in Highly Resistive Single Sb_2_Se_3_ Nanowires and Nanowire Bundles. *Sci. Rep*. **6**, 35086; doi: 10.1038/srep35086 (2016).

## Supplementary Material

Supplementary Information

## References

[b1] Rowe D. M. (ed.) CRC Handbook of Thermoelectrics, CRC, Boca Raton, (1995).

[b2] Matsubara K. Development of a high efficient thermoelectric stack for a waste exhaust heat recovery of vehicles Proceedings of International Conference on Thermoelectrics, 418–423 (2002).

[b3] Dresselhaus M. S. . New Directions for Low-dimensional Thermoelectric Materials. Adv. Mater. 19, 1043–1053 (2007).

[b4] Chen G., Dresselhaus M. S., Dresselhaus G., Fleurial J. P. & Caillat T. Recent Developments in Thermoelectric Materials. Int. Mater. Rev. 48, 45–66 (2003).

[b5] Hick L. D. & Dresselhaus M. S. Effect of Quantum-well Structures on the Thermoelectric Figure of Merit. Phys. Rev. B 47, 12727–12731 (1993). 10.1103/physrevb.47.12727 10005469

[b6] Hick L. D. & Dresselhaus M. S. Thermoelectric Figure of Merit of a One-dimensional Conductor. Phys. Rev. B 47, 16631–16634 (1993). 10.1103/physrevb.47.16631 10006109

[b7] Fardy M., Hochbaum A. I., Goldberger J., Zhang M. M. & Yang P. Synthesis and Thermoelectric Characterization of Lead Chalcogenide Nanowires. Adv. Mater. 19, 3047–3051 (2007).

[b8] Liang W. . Field-effect Modulation of Seebeck Coefficient in Single PbSe Nanowires. Nano Lett. 9, 1689–1693 (2009). 19309086 10.1021/nl900377e

[b9] Tai G., Zhou B. & Guo W. L. Structural Characterization and Thermoelectric Transport Properties of Uniform Single-Crystalline Lead Telluride Nanowires. J. Phys. Chem. C 112, 11314–11318 (2008).

[b10] Roh J. W. . Size-dependent Thermal Conductivity of Individual Single-crystalline PbTe Nanowires. Appl. Phys. Lett. 96, 103101 (2010).

[b11] Biswas K. . Strained Endotaxial Nanostructures with High Thermoelectric Figure of Merit. Nat. Chem. 3, 160–166 (2011). 21258390 10.1038/nchem.955

[b12] Androulakis J. . Spinodal Decomposition and Nucleation and Growth as a means to Built Nanostructured Thermoelectrics: Enhanced Performance in Pb_1−x_Sn_x_Te-PbS. J. Am. Chem. Soc. 129, 9780–9788 (2007). 17629270 10.1021/ja071875h

[b13] Hsu K. F. . Cubic AgPb_m_SbTe_2+m_: Bulk Thermoelectric Materials With High Figure of Merit. Science 303, 818–821 (2004). 14764873 10.1126/science.1092963

[b14] Kim P., Shi L., Majumdar A. & McEuen P. L. Thermal Transport Measurement of Individual Multiwalled nanotubes. Phys. Rev. Lett. 87, 215502 (2001). 11736348 10.1103/PhysRevLett.87.215502

[b15] Li D. . Thermal Conductivity of Individual Silicon Nanowires. Appl. Phys. Lett. 83, 2934–2936 (2003).

[b16] Fujii M. . Measuring the Thermal Conductivity of a Single Carbon nanotube. Phys. Rev. Lett. 95, 065502 (2005). 16090962 10.1103/PhysRevLett.95.065502

[b17] Shi L., Yao D., Zhang G. & Li B. Size Dependent Thermoelectric Properties of Silicon Nanowires. Appl. Phys. Lett. 95, 063102 (2009).

[b18] Roh J. W. . Size-dependent Thermal Conductivity of Individual Single-crystalline PbTe Nanowires. Appl. Phys. Lett. 96, 103101 (2010).

[b19] Doerk G. S., Carraro C. & Maboudian R. Single Nanowire Thermal Conductivity Measurements by Raman Thermography. ACS Nano 4, 4908–4914 (2010). 20731463 10.1021/nn1012429

[b20] Rojo M. M. . Decrease in Thermal Conductivity in Polymetric P3HT Nnaowires by Size-reduction Induced by Crystal Orientation: New Approaches Towards Thermal Transport Engineering of Organic Materials. Nanoscale 6, 7858–7865 (2014). 24933655 10.1039/c4nr00107a

[b21] Wrasse E. O., Torres A., Baierle R. J., Fazzio A. & Schmidt T. M. Size-effect Induced Thermoelectric Figure of Merit in PbSe and PbTe Nanowires. Phys. Chem. Chem. Phys. 16, 8114–8118 (2014). 24654001 10.1039/c3cp55233k

[b22] Platakis N. S. & Gatos H. C. Threshold and Memory Switching in Crystalline Chalcogenide Materials. Phys. Status Solidi A13, K1–K4 (1972).

[b23] Black J., Conwell E. M., Seigle L. & Spencer C. W. Electrical and Optical Properties of Some M_2_^V-B^N_3_^VI-B^ Semiconductors. J. Phys. Chem. Solids 2, 240–251 (1957).

[b24] Rajpure K. Y., Lokhande C. D. & Bhosele C. H. Effect of the Substrate Temperature on the Properties of Spray Deposited Sb-Se Thin Films from Non-aqueous Medium. Thin Solid Films 311, 114–118 (1997).

[b25] Nascimento V. B. . XPS and EELS Study of the Bismush Selenide. J. Electron Spectrosc. 104, 99–107 (1999).

[b26] Rajpure K. Y., Lokhande C. D. & Bhosale C. H. Photoelectrochemical Studies on Electrodeposited Cd-Fe-Se Thin Films. Mater. Res. Bull. 34, 1079–1087 (1999).

[b27] Zhai T., Li L. & Wang X. Recent Developments in One-dimensional Inorganic Nanostructures for Photodetectors. Adv. Funct. Mater. 20, 4233–4248 (2010).

[b28] Ma J. . One-dimensional Sb_2_Se_3_ Nanostructures: Solvothermal Synthesis, Growth Mechanism, Optical and Electrochemical Properties. CrysEngComm 13, 2369–2374 (2011).

[b29] Ma J. . Controlled Synthesis of One-dimensional Sb_2_Se_3_ Nanostructures and Their Electrochemical Properties. J. Phys. Chem. C 113, 13588–13592 (2009).

[b30] Choi D. . Diameter-controlled and Surface-modified Sb_2_Se_3_ Nanowires and Their Photodetector Performance. Sci. Rep. 4, 6714 (2014). 25336056 10.1038/srep06714 PMC4205837

[b31] Zhai T. . Single-crystalline Sb_2_Se_3_ Nanowires for High-performance Field Emitters and Photodetectors. Adv. Mater. 22, 4530–4533 (2010). 20836072 10.1002/adma.201002097

[b32] Ota J. & Srivastava S. K. Synthesis and Optical Properties of Sb_2_Se_3_ Nanorods. Opt. Mater. 32, 1488–1492 (2010).

[b33] Liu Y.-Q., Zhang M., Wang F.-X. & Pan G.-B. Facile Microwave-assisted Synthesis of Uniform Sb_2_Se_3_ Nanowires for High Performance Photodetectors. J. Mater. Chem. C 2, 240–244 (2014).

[b34] Chang H.-W., Sarkar B. & Liu C. W. Synthesis of Sb_2_Se_3_ Nanowires via a Solvothermal Route from the Single Source Precursor Sb[Se_2_P(O^i^Pr)_2_]_3_. Cryst. Growth Des. 7, 2691 (2007).

[b35] Lin Y.-F., Chang H.-W., Lu S.-Y. & Liu C. W. Preparation, Characterization, and Electrophysical Properties of Nanostructured BiPO_4_ and Bi_2_Se_3_ Derived from a Structurally Characterized, Single-Source Precursor Bi[Se_2_P(O*i*Pr)_2_]_3_. J. Phys. Chem. C 111, 18538 (2007).

[b36] Ko T. Y. . Electrical and Optical Properties of a Single Sb_2_Se_3_ Nanorod. Cent. Eur. J. Chem. 7, 197 (2009).

[b37] Shi L. . Measuring Thermal and Thermoelectric Properties of One-dimensional Nanostructures Using a Microfabricated Device. J. Heat Trans. –T ASME 125, 881–888 (2003).

[b38] Rojo M. M., Calero O. C., Lopeandia A. F., Rodriguez-Viejo J. & Martin-Gonzalez M. Review on Measurement Techniques of Transport Properties of Nanowires. Nanoscale 5, 11526–11544 (2013). 24113712 10.1039/c3nr03242f PMC4046576

[b39] Uphoff H. L. & Healy J. H. Thermoelectric Properties of Diphasal Systems Combining As_2_Te_3_ and Tl_2_Se with Sb_2_Te_3_, Bi_2_Te_3_, or Sb_2_Se_3_. J. Appl. Phys. 34, 390 (1963).

[b40] Mehta R. J. . High Electrical Conductivity Antimony Selenide Nanocrystals and Assemblies Nano Lett. 10, 4417–4422 (2010). 20925405 10.1021/nl1020848

[b41] Liu X. J., Zhang G. & Zhang Y. W. Tunable mechanical and thermal properties of one-dimensional carbine chain; phase transistion and microscopic dynamics. J. phys chem. C 119, 24156–24164 (2015).

[b42] Hochbaum A. I. . Enhanced thermoelectric performance of rough silicon nanowires. Nature 451, 163–167 (2008). 18185582 10.1038/nature06381

[b43] Lim J., Hippalgaonkar K., Andrews S. C., Majumdar A. & Yang P. Quantifying surface roughness effects on phonon transport in silicon nanowires. Nano Lett. 12, 2475–2482 (2012). 22524211 10.1021/nl3005868

[b44] Liu X. J., Zhang G., Pei Q. X. & Zhang Y. W. Modulating the thermal conductivity of silicon nanowires via surface amorphization. Sci. China Technol. Sci. 57, 699 (2014).

[b45] Abouelaoualim D. Size Effects on Nanowire Phonon Thermal Conductivity: a Numerical Investigation Using the Boltzmann Equation. Acta Phys. Pol. A 112, 49 (2007).

[b46] Prasher R. Acoustic Mismatch Model for Thermal Contact Resistance of van der Waals Contacts. Appl. Phys. Lett. 94, 041905 (2009).

[b47] Hu L. . Phonon Interference at Self-assembled Monolayer Interface: Molecular Dynamics Simulations. Phys. Rev. B 81, 235427 (2010).

[b48] Hone J., Whitney M., Piskoti C. & Zettl A. Thermal Conductivity of single-walled Carbon Nanotubes. Phys. Rev. B 59, R2514–R2516 (1999).

[b49] Seol J. H. . Two-dimensional Phonon Transport in supported graphene. Science 328, 213–216 (2010). 20378814 10.1126/science.1184014

[b50] Estrada D. & Pop E. Imaging Dissipation and Hot Spots in Carbon Nanotube Network Transistors. Appl. Phys. Lett. 98, 073102 (2011).

[b51] Jin Y. . Thermal Boundary Resistance of Copper Phthalocyanine-Metal Interface. Appl. Phys. Lett. 98, 093305 (2011).

[b52] Gilbert L. R., Van Pelt B. & Wood C. The Thermal Activation Energy of Crystalline Sb_2_Se_3_. J. Phys. Chem. Solids 35, 1629–1632 (1974).

[b53] Lin Y.-M., Rabin O., Cronin S. B., Ying J. Y. & Dresselhaus M. S. Semimetal-semiconductor Transition in Bi_1-x_Sb_x_ Alloy Nanowires and Their Thermoelectric Properties. Appl. Phys. Lett. 81, 2403–2405 (2002).

[b54] Pichard C. R., Tellier C. R. & Tosser A. J. Thermoelectric Power of Thin Polycrystalline Metal Films in An Effective Mean Free Path Model. J. Phys. F: Met. Phys. 10, 2009–2014 (1980).

