# Supplementary material for: Thermal and Thermoelectric Transport in Highly Resistive Single Sb2Se3 Nanowires and Nanowire Bundles
Source: Sci Rep. 2016 Oct 7;6:35086. doi: 10.1038/srep35086 (PMC5054389; doi:10.1038/srep35086)
Supplement: Supplementary Information [file srep35086-s1.pdf]

Supplementary Information

Thermal and Thermoelectric Transport in Highly Resistive Single Sb_2Se_3 Nanowires and Nanowire Bundles

Ting-Yu Ko [1], Muthaiah Shellaiah [1], and Kien Wen Sun [1,2,]*

[1]Department of Applied Chemistry, National Chiao Tung University, 1001 University Road, Hsinchu 30010, Taiwan

[2]Department of Electronics Engineering, National Chiao Tung University, 1001 University Road, Hsinchu 30010, Taiwan

E-mail: kwsun@mail.nctu.edu.tw

This document includes:

- Description of nanowire fabrication method
- Surface roughness analysis
- Thermal conductivity measurements on a single 120 nm – diameter NW

1. Fabrication of Sb_2Se_3 nanowires

1.1 Preparation of $Sb[Se_2P(O^iPr)_2]_3$ precursor

The synthesis of the $Sb[Se_2P(O^iPr)_2]_3$ precursor followed the modified procedure reported by Zingaro et al. [Inorg. Chem. 8, pp. 2337 (1969)] that had been used to prepare the $Sb[Se_2P(OEt)_2]_3$. A typical synthesis method of $NH_4[Se_2P(O^iPr)_2]$ compound is as follows: P_2Se_5 (2.96 g) was suspended in alcohol, then refluxed for 4 h until its color changed from colorless to yellowish during the reaction. The yellowish filtrate was kept in the icebath, then bubbled through with NH_3(g) until the solution became grayish. The solvent was evaporated under N_2, then a large amount of diethyl ether was added to afford white powder of $NH_4Se_2P(OR)_2$ (2.54 g). Thermally unstable, moisture and air-sensitive, colorless powders were obtained in 50–85% yields. A suspension of $NH_4[Se_2P(O^iPr)_2]$ (1.00 g, 3.076 mmol) in 40 mL of CH_2Cl_2 was added to $Sb(C_2H_3O_2)_3$ (0.306 g, 1.025 mmol), and the resulting mixture was stirred for 4 h under N_2 until the solution became yellowish. The reaction mixture was filtered through Celite under a N_2 atmosphere; the yellow filtrate was collected and evaporated to dryness using a rotary evaporator under reduced pressure. The resulting solid was dissolved in n-hexane (20 mL), filtered through Celite, and evaporated to dryness. After evaporation of the solvent, the yellow powder $Sb[Se_2P(O^iPr)_2]_3$ (0.984 g) was collected with 92% yields. A single crystal suitable for X-ray crystallography was grown from dichloromethane layered with hexane.

1.2 Nanowire synthesis via solvothermal method

The nanowires were prepared by a solvothermal method from the single-source precursor $Sb[Se_2P(OiPr)_2]_3$. $Sb[Se_2P(OiPr)_2]_3$ (800 mg, 0.767 mmol) was added to 15 mL of methanol and heated in a Teflon coated stainless steel autoclave at different temperature ranged from 100 ^oC to 200 ^oC for 12 h. After cooling to room temperature, the product appeared as a black solid. The solid was washed with methanol (1 mL x 3 times) and dried at 70 ^oC for 5 h. The composition of the solid was found by powder X-ray diffraction (XRD) and energy dispersive spectrometer (EDS) to be Sb_2Se_3.

2. Surface roughness of as-synthesized Sb_2Se_3 nanowires

Figure S1 displays the HRTEM image taken from the NW prepared at 100 ^{o}C. The mean surface roughness both at the center and edge of the NWs was less than 1 nm.

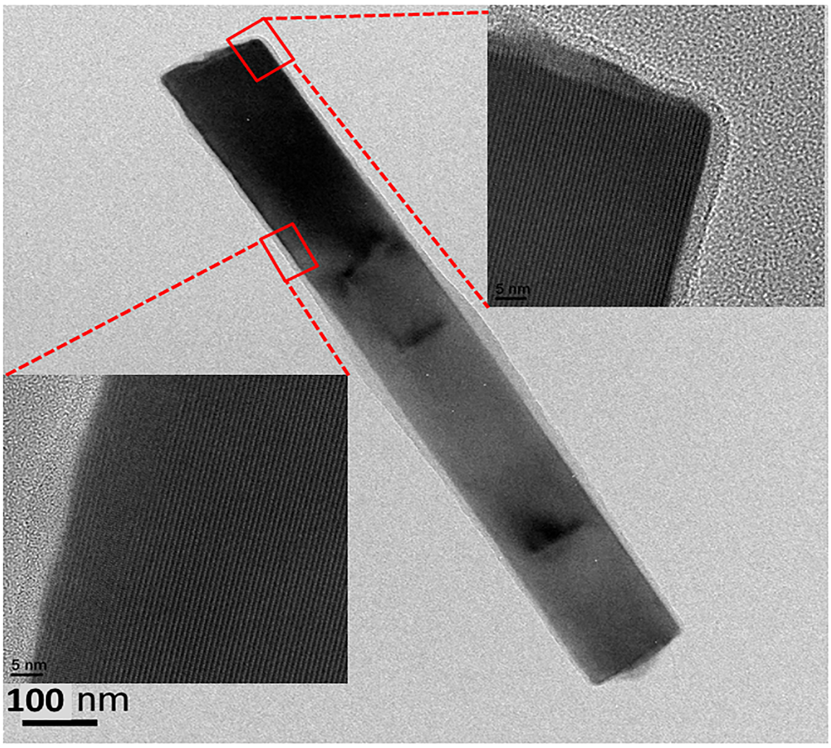

Figure S2 The HRTEM image taken from the NW prepared at 200 ^{o}C. The mean surface roughness was similar to that of NWs prepared at lower temperature.

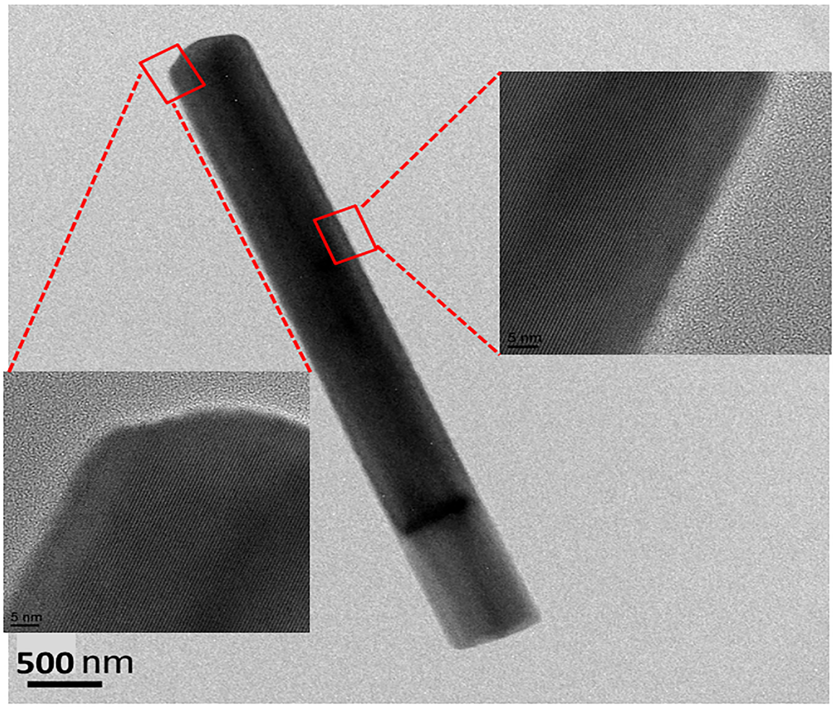

3. Thermal conductivity measurements of single Sb_2Se_3 120nm-diameter NW (NW D)

Figure S3 SEM image and instrumentation for measuring thermal conductivity of the NW D.

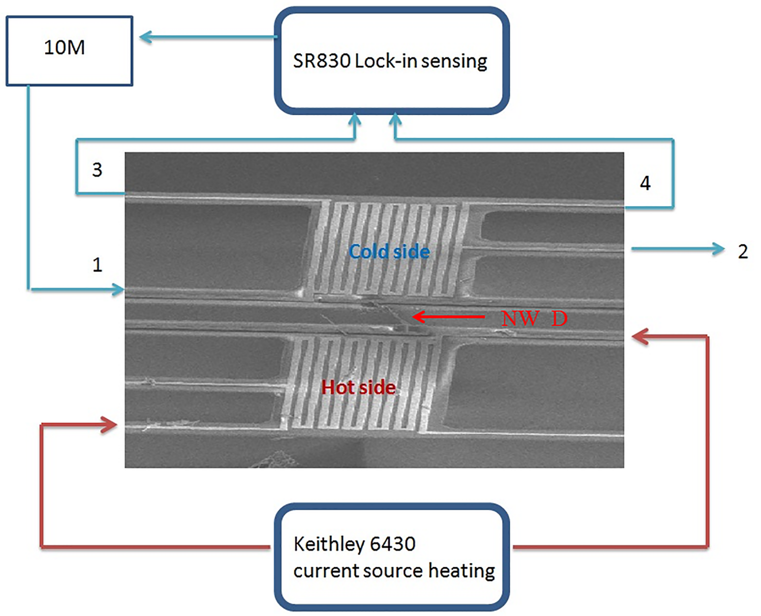

Figure S4 Changes in the sensing membrane temperature (ΔT_s) of NW A, NW B, NW C, and NW D as a function of joule heating power when an electrical current from 4 μA to 12 μA was applied to the heating membrane.

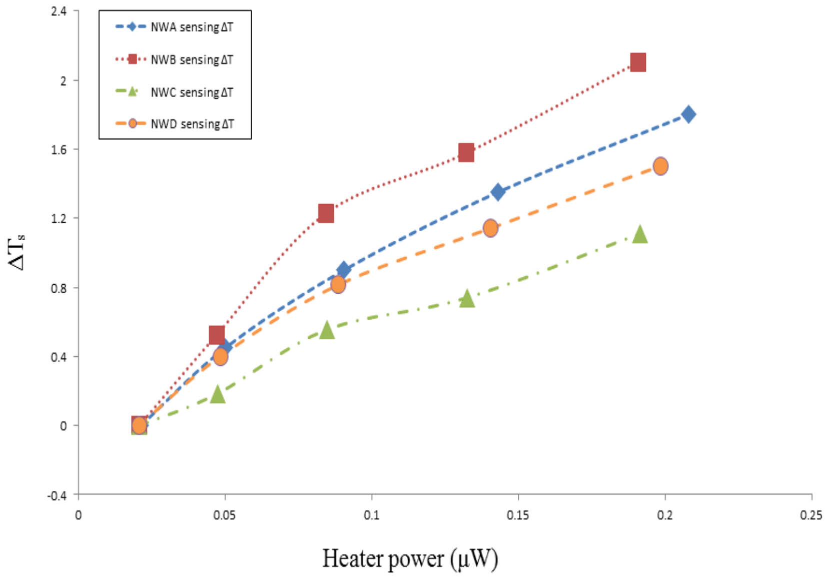

At a joule heat power of 4.67 x 10^{-8} W, the temperature changes on the heating and sensing

membranes for the NW D are $\Delta T_h = 4.95$ K and $\Delta T_s = 0.4$ K, respectively. Inputting these values in the equation $G_{NW} = G_b \frac{\Delta T_s}{\Delta T_h + \Delta T_s}$ and assuming that thermal contact resistance was negligible, we find that the thermal conductance of the NW D is $G_{NW} = 1.176 \times 10^{-9}$ W/K. This value is approximately 23% and 13% lower than that of NW B ($G_{NW} = 1.522 \times 10^{-9}$ W/K) and NW A ($G_{NW} = 1.311 \times 10^{-9}$ W/K), respectively.
